# Apoptosis Inducing Effects of Kuding Tea Polyphenols in Human Buccal Squamous Cell Carcinoma Cell Line BcaCD885

**DOI:** 10.3390/nu6083084

**Published:** 2014-08-05

**Authors:** Xin Zhao, Liang Pang, Jing Li, Jia-Le Song, Li-Hua Qiu

**Affiliations:** 1 Department of Biological and Chemical Engineering, Chongqing University of Education, Chongqing 400067, China; E-Mail: zhaoxin@pnu.edu; 2 Department of Oral and Maxillofacial Surgery, The Affiliated Hospital of Stomatology, Chongqing Medical University, Chongqing 401147, China; E-Mail: pangliang0221@gmail.com; 3 Department of Pharmacology, University of Illinois at Chicago, Chicago, IL 60607, USA; E-Mail: jingl@uic.edu; 4 Department of Food Science and Nutrition, Pusan National University, Busan 609-735, Korea; E-Mail: biopaul@pnu.edu

**Keywords:** Kuding tea, polyphenols, apoptosis, gene, human buccal squamous cell carcinoma cell line BcaCD885

## Abstract

Tea polyphenols are functional substances present in tea. Kuding tea as a traditional drink also contains these compounds. After 25, 50 and 100 μg/mL of Kuding tea polyphenol treatment for 48 h, cell proliferation of human buccal squamous cell carcinoma cell line BcaCD885 was inhibited, and the 100 μg/mL of Kuding tea polyphenol showed the highest inhibitory rate at 72.3%. Compared to the lower concentration, the 100 μg/mL of Kuding tea polyphenols significantly (*p* < 0.05) induced apoptosis as determined by flow cytometry analysis, the content of sub-G1 cancer cells was 32.7%. By RT-PCR and western blot assays, Kuding tea polyphenol significantly induced apoptosis in BcaCD885 cancer cells (*p* < 0.05) by upregulating caspase-3, caspase-8, caspase-9, Fas/FasL, Bax, p53, p21, E2F1, p73 and downregulating Bcl-2, Bcl-xL, HIAP-1, and HIAP-2 mRNA and protein expressions. Kuding tea polyphenols thus present apoptosis inducing effects *in vitro*.

## 1. Introduction

Kuding tea, as a type of traditional pure natural health beverage, is prepared from leaves of “Kudingcha”, a Holly, which is a type of Aquifoliaceae evergreen tree. It is mainly produced in the southwestern and southern region of China [1]. It contains more than 200 ingredients, which include Kuding saponin, amino acid, vitamin C, polyphenols, flavonoid, and protein, *etc.* [2]. According to traditional Chinese medicine, Kuding tea has the effects of lowering blood pressure, maintaining proper weight, and removing blood stasis. It also has the function of anti-cancer and anti-aging [3,4]. The polyphenols in tea are not only one of the dominant ingredients that constitute the color, aroma and taste of tea, but also one of the dominant ingredients for health care function. Some researches have shown that many such bioactive substances as tea polyphenols have the function of detoxification and anti-radiation [5]. It also has strong anti-cancer effect [6]. Although we call Kuding tea “tea beverage”, it is just a type of beverage similar to tea beverage because it is produced from different plants compared with traditional Chinese tea (such as green tea and black tea). In general, there are 20%–35% polyphenols in tea beverage [7]. However, some studies have shown that there are more than 10% polyphenols in Kuding tea [8].

Apoptosis is a type of programmed cell death implemented by cells using their own physiological and pathological factors. Physiological apoptosis can help to prevent illness. However, under the stimulation of functional substances, some genes of cancer cells may mutate or alter expression. Then the function, structure and growth state of cancer cells may change abnormally [9]. Many effective constituents in food can help to prevent cancer by stimulating cancer cells to induce apoptosis and then death [10]. As very important constituents of tea, polyphenols significantly contribute to apoptosis of cancer cells *in vitro*. And it has been proven that polyphenols, present in Kuding tea, are antioxidants [11]. However, the effects of these functional components against cancer need further studies. The present study starts by treating oral cancer cells *in vitro* with polyphenols extracted from Kuding tea. Then we will evaluate the role that these polyphenols plays in inducing apoptosis of human buccal squamous cell carcinoma cell line BcaCD885 *in vitro* and investigate the mechanism how these polyphenols fight against cancer cells by observing the influence of polyphenols on the growth of cancer cells and by checking the changes of apoptosis-inducing factors treated with polyphenols using RT-PCR and western blot.

## 2. Materials and Methods

### 2.1. Extraction of Kuding Tea Polyphenols

First, the leaves of Kuding tea were powdered after being frozen and dried and 30 g of the powder was put into 250 mL of distilled water and stirred at 90 °C. Then the Kuding tea was extracted for 1 h. After filtering, the filtrate was extracted for 2 h with 250 mL of acetic ether twice. The two organic phases were combined and dried with anhydrous sodium sulfate. Then acetic ether solvent was removed through depression and distillation. In the end, Kuding tea polyphenols were obtained as a yellow powder form. By the Folin-Ciocalteu method, the polyphenols content of Kuding tea was 16.7%.

### 2.2. Cancer Cell Preparation

Human buccal squamous cell carcinoma cell line BcaCD885 obtained from State Key Laboratory of Oral Diseases in Sichuan University (Chengdu, Sichuan, China) was used for this study. The cancer cells were cultured in RPMI-1640 medium (HyClone Cell Culture and Bioprocessing (Beijing), Beijing, China) supplemented with 10% FBS (HyClone) and 1% penicillin-streptomycin (HyClone) at 37 °C in a humidified atmosphere containing 5% CO_2_ (model 311 S/N29035; Forma, Waltham, MA, USA). The medium was changed every two days.

### 2.3. Growth Inhibition Measurement

Growth inhibitory effect of the Kuding tea polyphenol was measured by the trypan blue exclusion method. Human buccal squamous cell carcinoma cell line BcaCD885 cells were seeded in a 6-well plate at a density of 1 × 10^5^ cells/mL in a volume of 1 mL per well. After BcaCD885 cancer cell adherence for 24 h, the medium in the 6-well plate was discarded. Then Kuding tea polyphenol was mixed with RPMI-1640 medium, and the mixed 1 mL solution with concentrations of 25, 50 and 100 μg/mL Kuding tea polyphenol were added in 6-well plates and the cells were further incubated at 37 °C in 5% CO_2_ for 48 h. Then the BcaCD885 cells were stained with trypan blue solution and washed with phosphate-buffered saline (PBS), and counted using a hemocytometer [12].

### 2.4. Flow Cytometry Analysis

BcaCD885 cells were treated with 25, 50 and 100 μg/mL Kuding tea polyphenol under the same condition of growth inhibitory experiment. After treatment with Kuding tea polyphenol, the cells were trypsinized, collected, washed with cold phosphate-buffered saline (PBS), and resuspended in 2 mL PBS. DNA contents of the cells were measured using a DNA staining kit (CycleTEST™ PLUS kit; Becton Dickinson, Franklin Lakes, NJ, USA). Nuclear fractions stained with propidium iodide were obtained by following the manufacturer’s protocol. Fluorescence intensity was determined using a FACScan flow cytometer (EPICS XL-MCL; Beckman Coulter KK, Brea, CA, USA) and analyzed with CellQuest software (Becton Dickinson) [13].

### 2.5. mRNA Expression Measurement

Total RNA from human buccal squamous cells carcinoma cell line BcaCD885 was isolated using Trizol reagent (Invitrogen, Carlsbad, CA, USA) according to the manufacturer’s recommendations. The RNA was digested with RNase-free DNase (Roche, Basel, Switzerland) for 15 min at 37 °C and purified using an RNeasy kit (Qiagen, Hilden, Germany) according to the manufacturer’s protocol. cDNA was synthesized from 2 μg of total RNA by incubation at 37 °C for l h with avian myeloblastosis virus reverse transcriptase (GE Healthcare, Little Chalfont, United Kingdom) with random hexanucleotides according to the manufacturer’s instruction. Sequences of primers used to specifically amplify the genes of interest are shown in Table 1. Amplification was performed in a thermal cycler (Eppendorf, Hamburg, Germany). The polymerase chain reaction (PCR) products were separated in 1.0% agarose gels and visualized with ethidium bromide staining [14].

**Table 1:** Sequences of reverse transcription-polymerase chain reaction primers used in this study.

Gene name	Sequence
*Procapase-3*	Forward: 5′-CTG GAA TAT CCC TGG ACA AC-3′
	Reverse: 5′-CAG GTC AAC AGG TCC ATT TG-3′
*Procapase-8*	Forward: 5′-CCC CAC CCT CAC TTT GCT-3′
	Reverse: 5′-GGA GGA CCA GGC TCA CTT A-3′
*Procapase-9*	Forward: 5′-GGC CCT TCC TCG CTT CAT CTC-3′
	Reverse: 5′-GGT CCT TGG GCC TTC CTG GTA T-3′
*Bax*	Forward: 5′-AAG CTG AGC GAG TGT CTC CGG CG-3′
	Reverse: 5′-CAG ATG CCG GTT CAG GTA CTC AGT C-3′
*Bcl-2*	Forward: 5′-CTC GTC GCT ACC GTC GTG ACT TGG-3′
	Reverse: 5′-CAG ATG CCG GTT CAG GTA CTC AGT C-3′
*Bcl-xL*	Forward: 5′-CCC AGA AAG GAT ACA GCT GG-3′
	Reverse: 5′-GCG ATC CGA CTC ACC AAT AC-3′
*HIAP-1*	Forward: 5′-GCC TGA TGC TGG ATA ACT GG-3′
	Reverse: 5′-GGC GAC AGA AAA GTC AAT GG-3′
*HIAP-2*	Forward: 5′-GCC TGA TGC TGG ATA ACT GG-3′
	Reverse: 5′-GCT CTT GCC AAT TCT GAT GG-3′
*p53*	Forward: 5′-CTG AGG TTG GCT CTG ACT GTA CCA CCA TCC-3′
	Reverse: 5′-CTC ATT CAG CTC TCG GAA CAT CTC GAA GCG-3′
*p21*	Forward: 5′-GCA GAC CAG CAT GAC AGA TTT-3′
	Reverse: 5′-GGA TTA GGG CTT CCT CTT GGA-3′
*E2F1*	Forward: 5′-GGG GAG AAG TCA CGC TAT GA-3′
	Reverse: 5′-CTC AGG GCA CAG GAA AAC AT-3′
*p73*	Forward: 5′-GAC GGA ATT CAC CAC CAT CCT-3′
	Reverse: 5′-CCA GGC TCT CTT TCA GCT TCA-3′
*Fas*	Forward: 5′-GAA ATG AAA TCC AAA GCT-3′
	Reverse: 5′-TAA TTT AGA GGC AAA GTG GC-3′
*FasL*	Forward: 5′-GGA TTG GGC CTG GGG ATG TTT CA-3′
	Reverse: 5′-TTG TGG CTC AGG GGC AGG TTG TTG-3′
*GAPDH*	Forward: 5′-CGG AGT CAA CGG ATT TGG TC-3′
	Reverse: 5′-AGC CTT CTC CAT GGT CGT GA-3′

### 2.6. Protein Expression Measurement

Total cell lysates were obtained with an extraction buffer as previously described. Protein concentrations were determined using a protein assay kit (Bio-Rad, Hercules, CA, USA). For Western blot analysis, the cell lysates were separated by 12% SDS-PAGE, transferred into a polyvinylidene fluoride membrane (GE Healthcare), blocked with 5% skim milk, and incubated with the primary antibodies (1:1000 dilution). Antibodies against caspase-3, -8, -9, Bax, Bcl-2, Bcl-xL, HIAP-1, HIAP-2, p53, p21, E2F1 and p73 were obtained from Santa Cruz Biotechnology, Inc. (Santa Cruz, CA, USA). After incubation with the horseradish peroxidase-conjugated secondary antibody at room temperature, immunoreactive proteins were detected using a chemiluminescent enhanced chemiluminescence assay kit (GE Healthcare) according to the manufacturer’s instructions. Bands in the blot were visualized using a LAS3000 luminescent image analyzer (Fujifilm Life Science, Tokyo, Japan) [15].

### 2.7. Statistical Analysis

Data are presented as the mean ± SD. Differences between the mean values for individual groups were assessed using one-way ANOVA followed by Duncan’s multiple range tests. Differences were considered significant when *p* < 0.05. SAS version 9.1 (SAS Institute Inc., Cary, NC, USA, 2009) was used for statistical analyses [15].

## 3. Results

### 3.1. Growth Inhibitory Effects of Kuding Tea Polyphenol against BcaCD885 Cells

**Figure 1:**
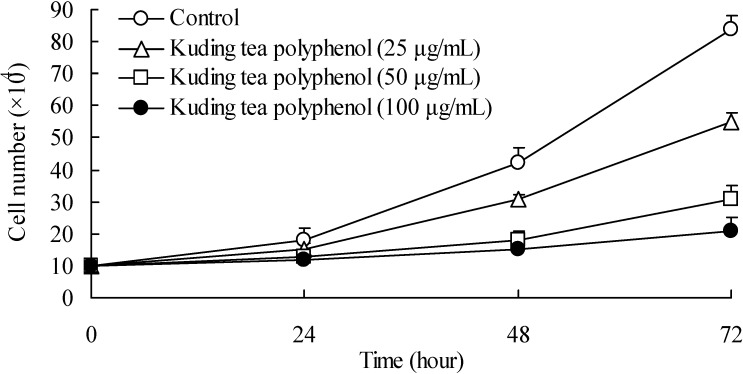
Time-dependent growth inhibition by Kuding tea polyphenol in human buccal squamous cell carcinoma cell line BcaCD885. Cells were plated at an initial density of 1 × 10^5^ cells per plate in six well plates and incubated for 24 h.

The growth inhibitory effects assessed by hemocytometer counts (cytotoxicity) for the cancer cells were determined (Figure 1). BcaCD885 cells were treated with 25, 50 and 100 μg/mL Kuding tea polyphenol for up to 3 days. At the first day, the different concentration of Kuding tea polyphenol showed similar cell growth numbers as the control, but after 2 days of incubation, growth of treated cells was gradually inhibited in a concentration-dependent manner. After 4 days, the 100 μg/mL Kuding tea polyphenol treated cancer cells were markedly inhibited compared to control cells; 25 and 50 μg/mL Kuding tea polyphenol also had inhibitory effect, but weaker than the 100 μg/mL Kuding tea polyphenol treatment. After 48 h Kuding tea polyphenol treatment, the growth of cancer cells was obviously inhibited. At a concentration of 100 μg/mL, 72 h treated cells were almost dead, thus the treatment time of 48 h was chosen to do other experiments in this study.

### 3.2. Induction of Apoptosis by Kuding Tea Polyphenol

**Figure 2:**
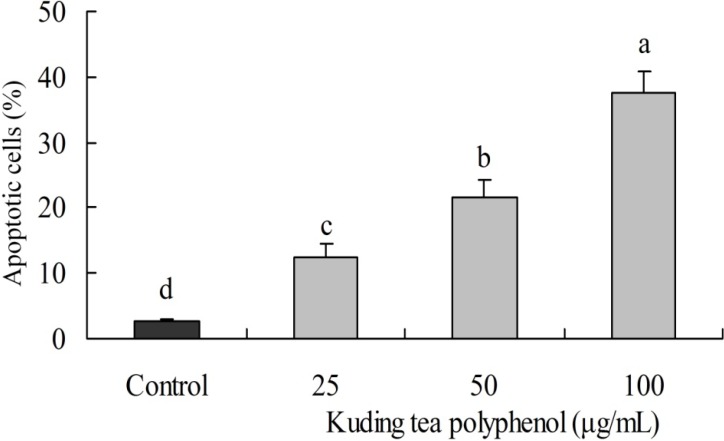
Apoptosis induced by Kuding tea polyphenol. The DNA content of sub-G1 human buccal squamous cell carcinoma cell line BcaCD885 was evaluated using a flow cytometer. Apoptosis was monitored by staining with annexin-V FITC. **a**–**d**, mean values with different letters over the bars are significantly different (*p* < 0.05) according to Duncan’s multiple range test.

DNA content of the sub-G1 BcaCD885 cancer cells was evaluated by flow cytometric analysis. The induction of apoptosis was almost negligible at 2.70% of sub-G1 content in control cancer cells. However, cancer cells treated with 100 μg/mL Kuding tea polyphenol had a higher level of apoptosis (37.6%) than those treated with 25 and 50 μg/mL Kuding tea polyphenol at 12.3% and 21.6%, respectively (Figure 2).

### 3.3. Gene Expression of the Apoptosis-Related Procapases and Fas/FasL

**Figure 3:**
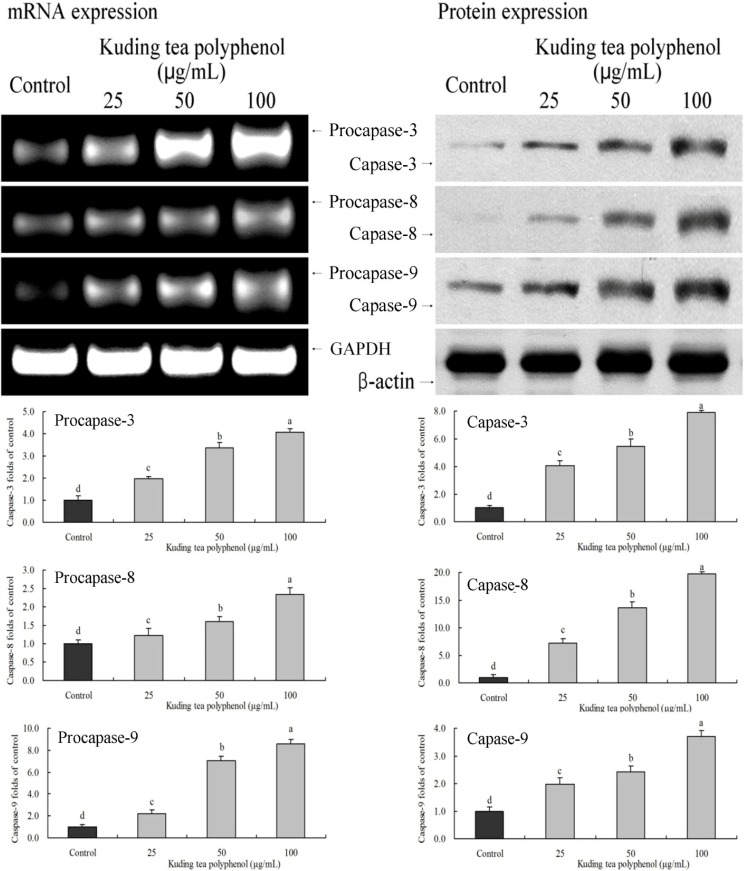
Effects of Kuding tea polyphenol on the mRNA expression of procapase-3, -8, -9 and protein expression of capase-3, -8 , -9 in human buccal squamous cell carcinoma cell line BcaCD885. Fold-ratio: gene expression/GAPDH (β-actin) × control numerical value (control fold ratio: 1). **a**–**d**, mean values with different letters over the bars are significantly different (*p* < 0.05) according to Duncan’s multiple range test.

The expression of procapase-3, -8 and -9 and capase-3, -8 and -9 in BcaCD885 cancer cells was determined by RT-PCR or western blotting after a 48-h incubation with 25, 50 and 100 μg/mL Kuding tea polyphenol. As shown in Figure 3, treatment with Kuding tea polyphenol markedly altered the levels of procapase and capase genes. Kuding tea polyphenol could increase the expression level of these genes. The higher concentration of Kuding tea polyphenol showed the more obvious increase.

**Figure 4:**
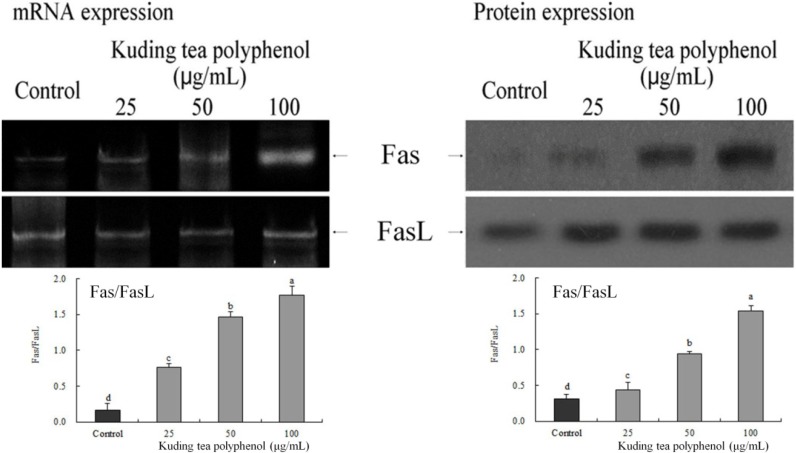
Effects of Kuding tea polyphenol on the mRNA and protein expression of Fas/FasL in human buccal squamous cell carcinoma cell line BcaCD885. **a**–**d**, mean values with different letters over the bars are significantly different (*p* < 0.05) according to Duncan’s multiple range test.

As shown in Figure 4, the expression levels of Fas depended on the increase of treated concentration of Kuding tea polyphenol, but expression levels of FasL did not exhibit any such differences between different concentration treatments. The value of Fas/FasL both increased with higher concentrations of Kuding tea polyphenol.

### 3.4. Gene Expression of the Apoptosis-Related Bcl-2 Family

**Figure 5:**
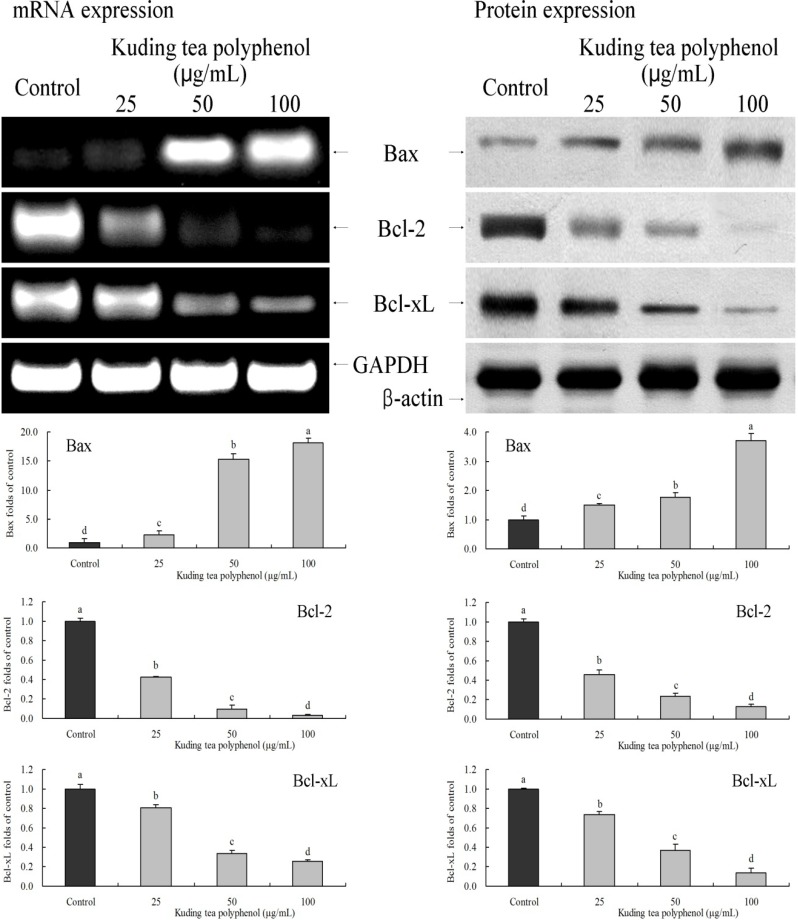
Effects of Kuding tea polyphenol on the mRNA and protein expression of Bax, Bcl-2 and Bcl-xL in human buccal squamous cell carcinoma cell line BcaCD885. Fold-ratio: gene expression/GAPDH (β-actin) × control numerical value (control fold ratio: 1). **a**–**d**, mean values with different letters over the bars are significantly different (*p* < 0.05) according to Duncan’s multiple range test.

Bcl-2 family genes are important apoptosis-related genes. Kuding tea polyphenol significantly (*p* < 0.05) changed the expression of Bax, Bcl-2 and Bcl-xL. Protein and mRNA expression levels of Bax were increased by Kuding tea polyphenol treatment, Bcl-2 and Bcl-xL expression showed opposite trends (Figure 5).

### 3.5. Gene Expression of the Apoptosis-Related HIAP-1 and HIAP-2

**Figure 6:**
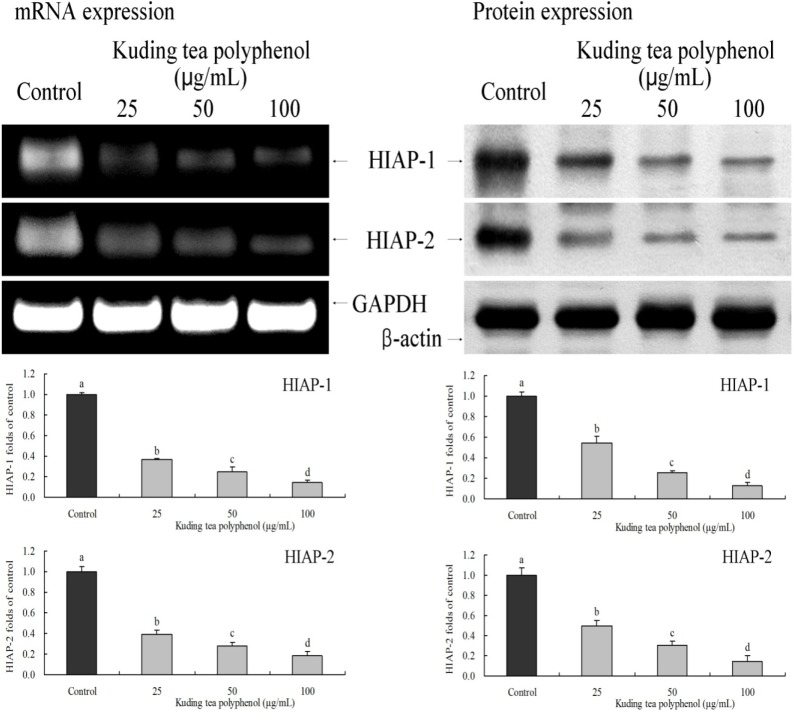
Effects of Kuding tea polyphenol on the mRNA and protein expression of HIAP-1 and HIAP-2 in human buccal squamous cell carcinoma cell line BcaCD885. Fold-ratio: gene expression/GAPDH (β-actin) × control numerical value (control fold ratio: 1). **a**–**d**, mean values with different letters over the bars are significantly different (*p* < 0.05) according to Duncan’s multiple range test.

HIAP-1 and HIAP-2 mRNA and protein expression levels of BcaCD885 cancer cells decreased when BcaCD885 cells were treated with Kuding tea polyphenol (Figure 6); the expression levels in the highest concentration of 100 μg/mL treated cancer cells were significantly (*p* < 0.05) lower than those with 25 and 50 μg/mL Kuding tea polyphenol treatment.

### 3.6. Gene Expression of the Apoptosis-Related p53 and p21

**Figure 7:**
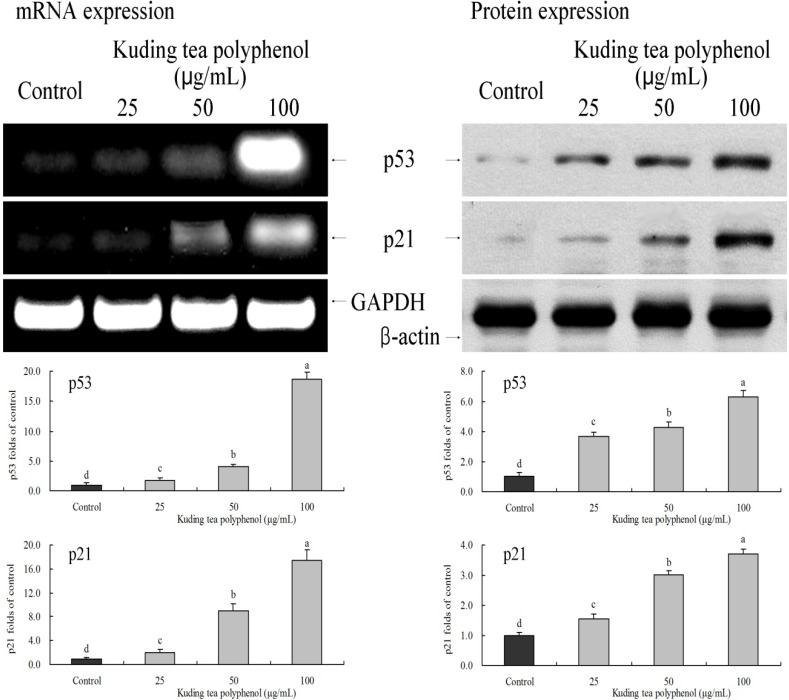
Effects of Kuding tea polyphenol on the mRNA and protein expression of p53 and p21 in human buccal squamous cell carcinoma cell line BcaCD885. Fold-ratio: gene expression/GAPDH (β-actin) × control numerical value (control fold ratio: 1). **a**–**d**, mean values with different letters over the bars are significantly different (*p* < 0.05) according to Duncan’s multiple range test.

After treatment with 100 μg/mL Kuding tea polyphenol, the mRNA and protein expression levels of p53 were 18.7 and 6.3 times higher than that of control cells which were untreated with Kuding tea polyphenol. p21 expression levels were also significantly increased, 17.5 and 3.7 times higher than control cancer cells. The 25 and 50 μg/mL Kuding tea polyphenol treatments also increased the expression of p53 to about 1.7–4.2 times that of control cells (Figure 7).

### 3.7. Gene Expression of the Apoptosis-Related E2F1 and p73

**Figure 8:**
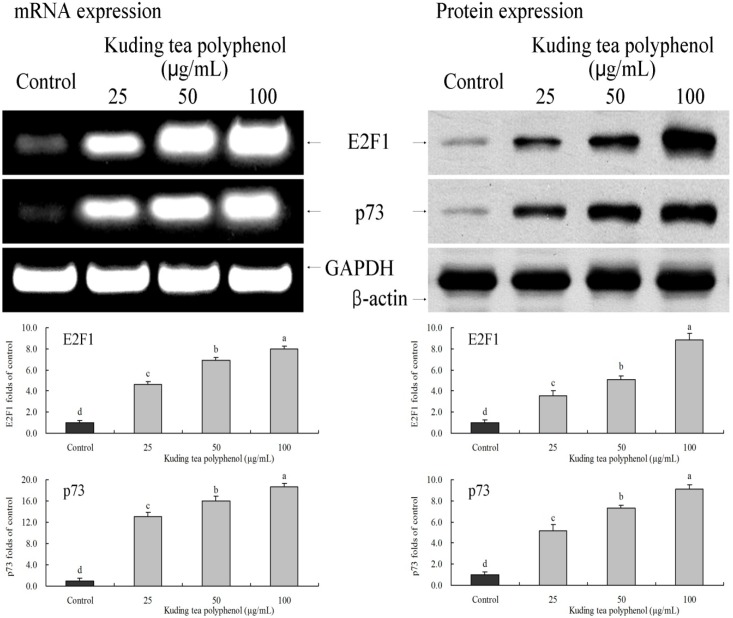
Effects of Kuding tea polyphenol on the mRNA and protein expression of E2F1 and p73 in human buccal squamous cell carcinoma cell line BcaCD885. Fold-ratio: gene expression/GAPDH (β-actin) × control numerical value (control fold ratio: 1). **a**–**d**, Mean values with different letters over the bars are significantly different (*p* < 0.05) according to Duncan’s multiple range test.

E2F1 and p73 expressions of Kuding tea polyphenol treated cancer cells were checked by RT-PCR and western blot experiments (Figure 8). E2F1 and p73 mRNA and protein expression levels in 100 μg/mL Kuding tea polyphenol treated BcaCD885 cancer cells were higher than those in 25 and 50 μg/mL Kuding tea polyphenol treated cells. Also, E2F1 and p73 expression levels in the three concentrations of Kuding tea polyphenol treated cells were increased compared to those levels in untreated control cancer cells.

## 4. Discussion

The main anti-cancer components in tea are tea catechin-dominant polyphenols. Since Kuding tea can inhibit cancer cells *in vitro*, the main functional components may also be polyphenols. The present study examined the anti-cancer effects of Kuding tea polyphenols by observing their influence on gene expression of BcaCD885 cancer cells.

Caspases are a type of protease hydrolysate that usually exists in the form of procaspases. However, caspase-3, -8 and -9 are the main protease hydrolysates involved in the process of apoptosis among these procaspases [16]. Caspase-8 and -9 are upstream caspases. They are apoptosis-triggering caspases that are responsible for activating downstream caspases. Caspase-3 is a downstream caspase. They are apoptosis-effecting caspases responsible for hydrolyzing apoptosis-effector molecules [17]. Both activation of caspase-8 induced by death receptor-mediated apoptotic pathways and activation of caspase-9 induced by mitochondria-mediated apoptotic pathways can trigger downstream caspase cascade reaction. Hydrolysis activates caspase-3 and this active caspase induces apotosis. When caspases trigger apoptosis, proteins such as calpain, cathepsin and endonucleases will carry out programmed cell death. Activatory caspase-9 is dependent upon cytochrome c, and Bcl-2 is localized to the mitochondrial membrane, Bcl-2 could prevent release of cytochrome c [18]. By interacting with caspase, these proteins help accelerate apoptosis. Kuding tea polyphenols can strengthen the expression of caspase-3, and induce apoptosis of BcaCD885 cells.

Fas could form the death-inducing signaling complex upon ligand binding. Active caspase-8 is then released from the death-inducing signaling complex into the cytosol, where it cleaves other effector caspases, which eventually leads to DNA degradation, membrane blebbing, and other hallmarks of apoptosis [19]. According to a new study, the extrinsic Fas pathway is sufficient to induce complete apoptosis in certain cell types and subsequent caspase-8 activation [20]. Apoptosis via the Fas/FasL system is necessary to decrease the activated lymphocyte population in the periphery after the immune response has killed off the pathogen [21]. To block apoptosis, the parasite in its amastigote stage blocks caspase-8, so the apoptotic signal delivered by Fas/FasL binding is arrested [22]. Kuding Tea polyphenols could increase the Fas/FasL value. By this way, Kuding Tea polyphenols might show a strong anticancer effect.

Bcl-2 and Bcl-xL are inhibitors of apoptosis proteins. However, Bax is a pro-apoptotic protein, and the main apoptosis molecule in the Bcl-2 family. When Bax is combined with Bcl-2 and Bcl-xL, it inactivates Bcl-2 and Bcl-xL [23]. Once Bcl-2 and Bcl-xL are neutralized and weakened, active Bax will transpose to mitocondria and stimulate mitochondrial outer membrane permeabilization (MOMP) to release pro-apoptotic molecules into proteins, thus inducing apoptosis of cancer cells. In addition, MOMP can also be stimulated when Bax is activated by some functional components directly, thus inducing apoptosis of cancer cells [24]. Kuding tea polyphenols are able to up-regulate Bax expression and the higher the level of Kuding tea polyphenols, the more obvious the up-regulation of expression is, thus inducing stronger apoptosis.

IAP, which includes HIAP-1 and HIAP-2, is a natural inhibitor of caspase in cells. Both HIAP-1 and HIAP-2 contain three BIR structural domains, which are necessary components of IAP in inhibiting apoptosis. Besides BIR structural domains, HIAP-1 and HIAP-2 also contain a CARD structural domain, which is a type of effector domain that combines death receptors competitively to inhibit the activation of caspase proenzymes, thus inhibiting apoptosis [25]. Therefore, if natural HIAP-1 and HIAP-2 in cancer cells can be controlled and weakened, activation of caspases will be reduced and caspase will fully play its role of apoptosis of cancer cells. By combining the result of the experiment of the expression of caspases, we can see that Kuding tea polyphenols can weaken the expression of HIAP-1 and HIAP-2, thus strengthening the expressions of caspases [26]. HIAP 1 and HIAP-2 could suppress TNF-receptor signaling by binding to the TNF receptor-associated factor, and Xiap suppresses apoptosis via caspase-3 inhibition [27].

p53 is a anti-oncogene which is most highly involved in cancer. p53 participates in the regulation and control of the cell cycle and repair of damaged DNA. Once DNA cannot be repaired, p53 will induce apoptosis of cancer cells. As a type of transcription factor, p53 can stimulate up-regulation of the expressions of Bax and participate in the process of mitochondria-mediated apoptosis through Bax [28]. In addition, p53 is able to combine with Bcl-xL and release Bax from compounds of Bax/Bcl-xL, thus inducing oligomerization. Oligomeric Bax can release some pro-apoptotic proteins from the mitochondria into thecytoplasm where caspase effector molecules will be activated, thus inducing apoptosis [29]. Lose of p53 impairs caspase-dependent apoptosis of the mitochondrial pathway, and caspase-9 is an important target for this development [30]. Under some conditions, p21 can promote apoptosis. According to some researchers, the expression of p21 is closely related to the proapoptotic protein Bax during the process of apoptosis. When the expression of Bax increases, the expression of p21 will also increase [31]. Moreover, the role that p21 plays in apoptosis is greater than the role Bcl-2 plays in inhibiting apoptosis.

E2F1 is able to induce apoptosis specifically. High expression of E2F1 can stimulate cell cancer cells to enter S phase and induce apoptosis, and can also strengthen the ability of p53 in inducing apoptosis by increasing the stability of p53 or stimulating p53 directly [32]. In addition, high expression of E2F1 can also induce apoptosis through p73. p73 genes can be activated by transcription factor E2F1 independently and induce apoptosis without the help of p53 [33].

Tea has a strong anticancer effect, the most important functional content of tea are polyphenols. Researcher found that tea polyphenols induced apoptosis and cell cycle arrest in human cancer cells. It is important that the apoptotic response of tea polyphenols were specific to cancer cells, but not in normal human epidermal keratinocytes [6].

## 5. Conclusions

This study treated cancer cells *in vitro* with extracted Kuding tea polyphenols, and growth rate of cells as well as mRNA and protein expression of apoptosis-related genes in BcaCD885 cancer cells was then investigated. The study concludes that Kuding tea polyphenols are able to inhibit cancer cells growth, slowing down BcaCD885 cell proliferation, upregulate the expression of caspase-3, caspase-8, caspase-9, Fas/FasL, Bax p53, p21, E2F1, p73 and down-regulate the expression of Bcl-2, Bcl-xL, HIAP-1, and HIAP-2. The results of our experiments confirm that Kuding tea polyphenols are able to inhibit cancer cell growth by inducing apoptosis of BcaCD885 cancer cells. The anticancer effect of Kuding tea polyphenols thus calls for further in vivo tests, and for the dose concentrations and mechanism to be determined.
